# Cathrombosis: Deep Vein Thrombosis After a Cat Bite - A Case Report

**DOI:** 10.7759/cureus.4924

**Published:** 2019-06-17

**Authors:** Francisco Guadarrama-Conzuelo, Alejandro Gutierrez-Castillo

**Affiliations:** 1 Escuela De Medicina, Instituto Tecnológico Y De Estudios Superiores De Monterrey, Monterrey, MEX; 2 Miscellaneous, Instituto Tecnológico Y De Estudios Superiores De Monterrey, Monterrey, MEX

**Keywords:** cat, deep vein thrombosis, animal bites, case report

## Abstract

Cat bites represent between 5-15% of all animal bites and are sometimes encountered by primary care and emergency department physicians. Other than polymicrobial infections, very few other complications have been reported.

We present the case of a 75-year-old male who developed deep vein thrombosis (DVT) in his left leg where he had been bitten by a cat four days prior. Although it is not possible to describe a causal relationship, we discuss whether these events are related.

## Introduction

For thousands of years, cats have been worshiped, cherished, and admired. Once living in the wild, they have now become a popular companion for humans [1]. Members of the *Felis catus* species meow, purr, and sleep. And sometimes, they bite.

Animal bites are a habitual problem commonly encountered by primary care physicians and in the emergency departments, as they are thought to account for approximately 1% of all ED consultations with over 4.5 million annual visits in the United States [2]. Cat bites represent 5-15% of the total [3], although this figure could be the consequence of under-reporting, as bites made by domestic cats are often considered by patients to be of inconsequential nature [4].

Cat bite demographics show that they are more commonly found in women than in men and in older adults, particularly those over 75 years old [3-4]. Cat bites are more frequent in the mornings, in the summer, and in spring [4]. They are most often located on the upper extremities and are almost always accompanied by scratches [2-4]. Cat bites are typically puncture wounds that penetrate deeply into the skin and underlying tissues [2] with infection rates of up to 50% [4]. Attackers are usually stray females [3].

Other than direct tissue damage by the bite itself and subsequent infections with varying degrees of severity [5], very few other complications of cat bites have been reported [6-7]. Here, we present a case in which deep vein thrombosis (DVT) occurred four days after a cat bite, and then we discuss whether these events are related.

## Case presentation

A 75-year-old male arrived to the ED after having experienced increasing pain and abnormal swelling of his left leg. Further questioning revealed a previous history of an attack by a vaccinated male cat that included bites and scratches in the left calf, four days prior. The patient referred that the attack produced three superficial puncture wounds, involving the upper dermis, that received adequate wound management with bi-daily cleaning with saline irrigation and change of dressings. No antibiotic prophylaxis or analgesics were prescribed. Despite no signs of systemic infection or changes in skin color, texture or temperature, the sudden increase in pain and swelling at the wound region led the patient to seek further medical attention. Past medical history was relevant only for primary hypertension, diagnosed about 15 years earlier and without treatment for the previous five years, allergy to clindamycin, and an inguinal hernioplasty four years prior. No personal history of cancer or hemostasis disorders was reported.

At admission, his vital signs were within normal limits. Upon examination, unilateral pitting edema and weakened tibial pulses were found in his left leg. No changes in skin color or warmth suggestive of cellulitis were detected. Calf diameter was different between the two legs (>3 cm). No Homan’s sign, tachypnea or abnormal heart or lung sounds were found. The patient did not report chest pain or dyspnea. The modified Wells’ score for deep-vein thrombosis was three points.

**Figure 1:**
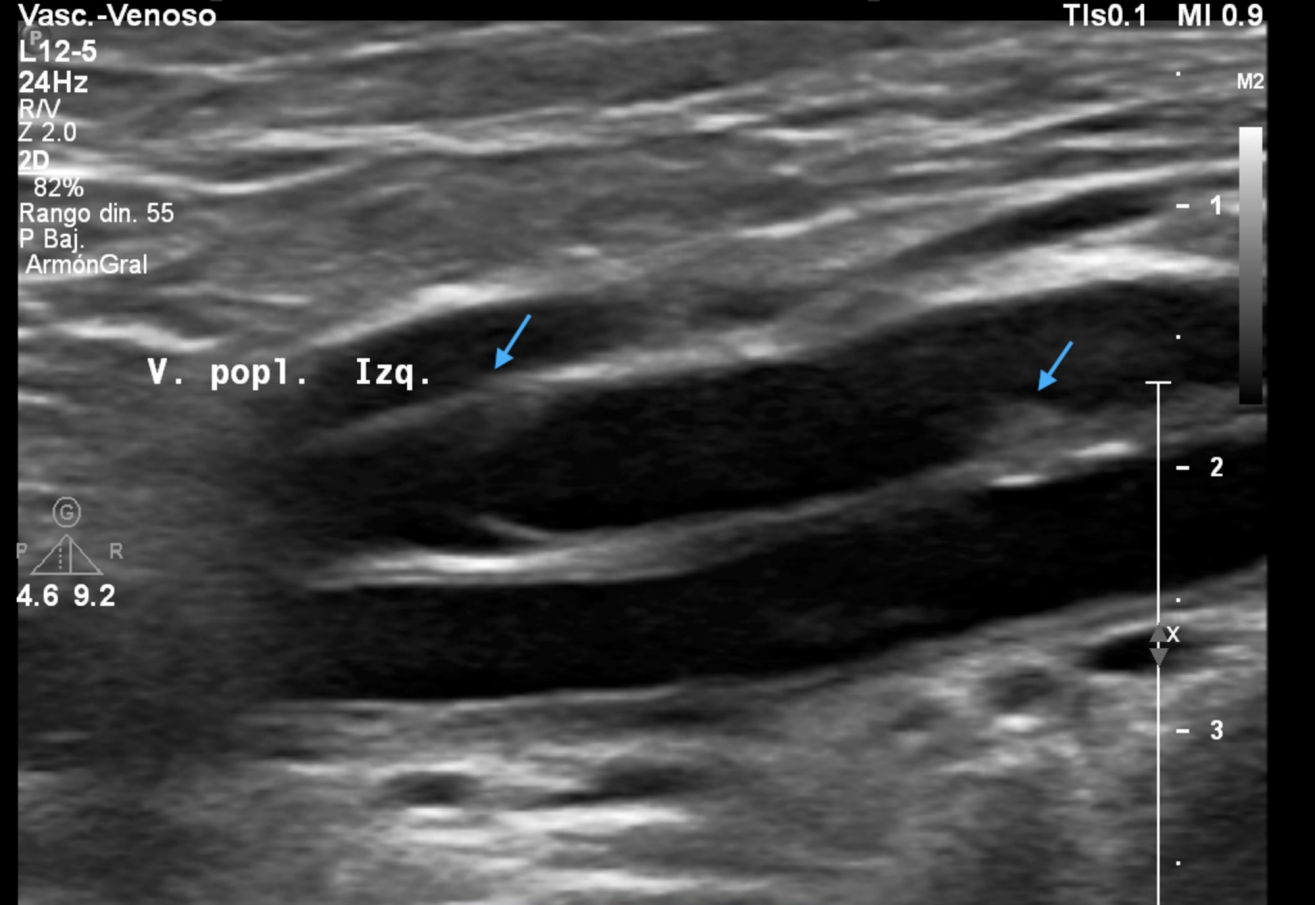
Deep vein thrombosis of the left popliteal vein Sonogram of the left popliteal vein (V. popl. Izq.) showing thrombi (blue arrows).

A compression venous ultrasound with Doppler imaging of the left leg revealed an echogenic non-compressible subvalvular thrombus on the middle portion of the popliteal vein. No other thrombi were detected (Figure 1).

The diagnosis of DVT was made and laboratory tests were ordered to investigate potential causes: complete blood count (CBC), coagulation profile, prostate-specific antigen, comprehensive metabolic panel, HbA1c, fecal occult blood test, D-dimer, and urinalysis were all within reference parameters. No additional imaging studies were ordered.

The patient’s clinical history, exhaustive physical exam, and laboratory evaluations showed no signs of underlying malignancy, autoimmune disease or infection. As no clear cause was established, and complying with the patient’s request, outpatient management for DVT was initiated with subcutaneous enoxaparin (1 mg/kg) twice daily and the usual non-pharmacological recommendations. The patient was told to come for a follow-up sonogram 10 days after starting therapy and to discuss the transition to oral medication.

**Figure 2:**
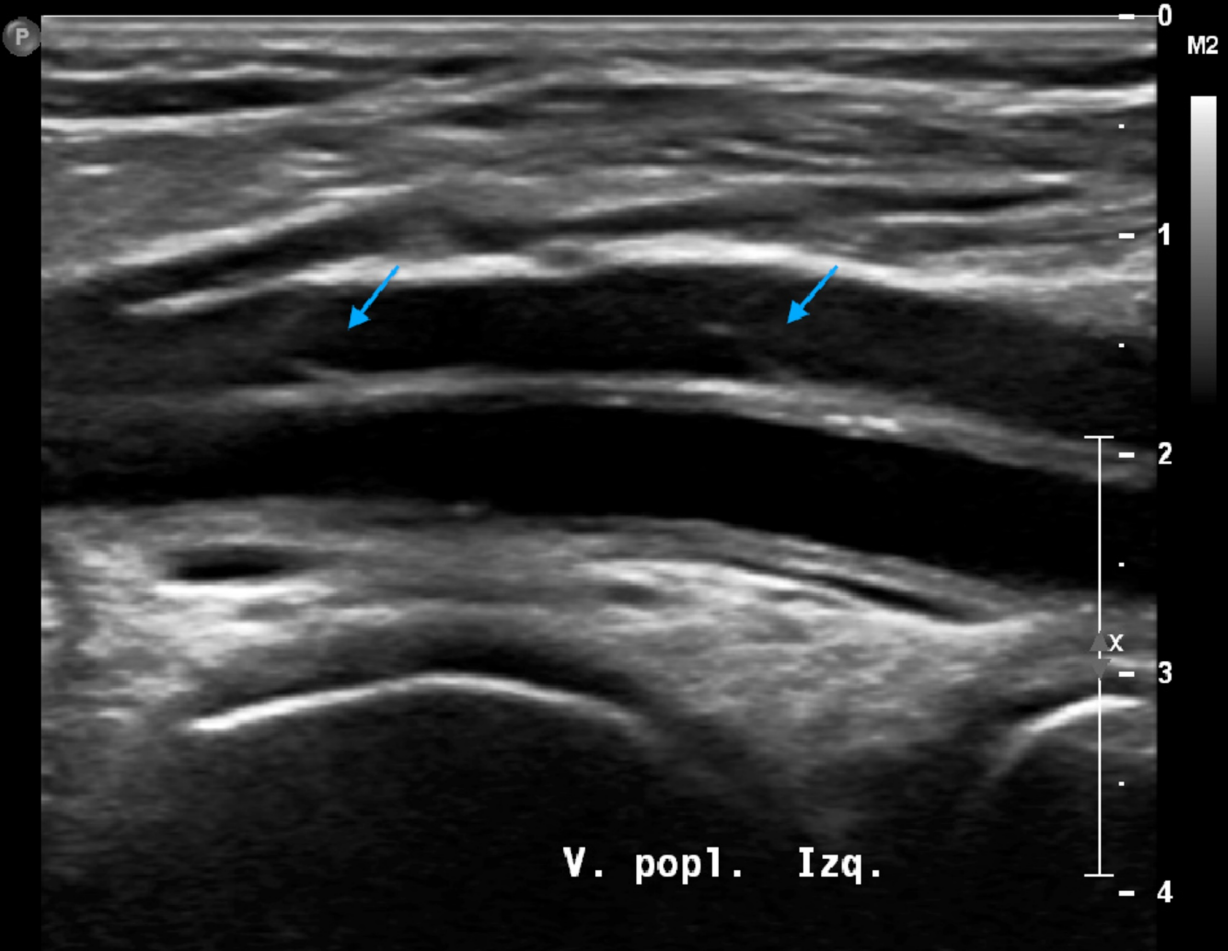
Sonogram of the left popliteal vein taken 13 days after the first one A clean venous lumen with normal valvular function can be seen (blue arrows).

The succeeding ultrasound - performed 13 days after the initial diagnosis - showed an anechoic lumen with complete remission and normal venous valvular function (Figure 2). As the initial parenteral therapy with low-molecular-weight heparin (LMWH) had been completed, treatment was changed to 15 mg rivaroxaban twice daily for 21 days and then 20 mg once daily for three to six months. Four months after the events here reported, the patient remains asymptomatic and is being treated with amlodipine 5 mg/losartan 100 mg QD and rivaroxaban 20 mg once daily.

## Discussion

Animal bites are more commonly caused by dogs, who are responsible for about 85-90% of all animal bites, but bites made by cats are second in frequency [2-4, 8], and thus are clinically relevant. Once a patient has been bitten by a cat, adequate cleansing and wound exploration are imperative. Generous irrigation with normal saline, chlorhexidine-based or povidone-iodine solutions must be done in a fashion that ensures high pressure [3-4, 8]. Once cleansing is completed, special care must be put in exploring the wound, as it is not uncommon to find foreign bodies (sometimes, teeth from the feline attacker). If damage to tendon, joint, or bone is suspected, specialized consultation must be sought.

If the wound is deemed to have spared deep structures, cleansing, exploration, and debridement are usually enough. Data on antibiotic prophylaxis for cat bites are scarce [2-4] and conflicting [8], but general agreement exists in favor of prescribing a short course of antibiotics, as the bites are considered to be high-risk. The usual choice is amoxicillin/clavulanate 875/125 mg every 12 hours for three to seven days [2, 8]. When the wound caused by a cat bite does become infected, cultures are often polymicrobial in nature but the most commonly found aerobic microorganism is *Pasteurella multocida*, followed by isolates of the genera *Streptococcus* and *Staphylococcus*. Frequent anaerobic bacteria isolates include *Fusobacterium spp.*, *Porphyromonas spp.*, and *Bacteroides spp. *[9]. Other infections known to be caused by cats are those caused by *Bartonella henselae*, *Francisella tularensis, Yersinia pestis *[10], *Sporothrix schenckii *[11], *Capnocytophaga canimorsus*, *C canis*, *C cynodegmi *[12] , and *Rickettsia felis *[13].

Other than direct tissue damage by the bite itself and subsequent infections with varying degrees of severity, very few other complications of cat bites have been reported, like subcutaneous emphysema and fetal tachycardia [6-7]. Although no causal relationship can be established between a cat bite and DVT, it is remarkable that this case occurred only four days after the attack. Previous evidence exists of a fatal pulmonary thromboembolism secondary to cellulitis caused by a dog bite [14], but, to our knowledge, no previous reports exist on the potential association between cat bites and DVT.

As a matter of fact, up to 50% of all episodes of DVT do not have a clearly identifiable cause [15-16]. DVT is more commonly found in the legs, in black people and in males (when pregnant women and women utilizing exogen estrogens are excluded) [15]. The causative mechanism for DVT is not completely understood, but it is pertinent to remember that for a thrombus to occur, Virchow’s triad states that three factors must be present: endothelial dysfunction or injury, hemodynamic changes (either stasis or turbulence) and hypercoagulability. In this case, older age and potential dehydration contributed to hypercoagulability; vascular damage was inflicted by the bite itself and by the chronic inflammatory state caused by untreated hypertension. Turbulent flow could have been caused by trauma.

Previous statements exist of the association between infections and thrombosis [17], but in the case here reported there was no clinical or laboratory evidence that documented local or systemic infections. An area of future research in cat-related DVT is the potential presence of thrombus-inducing proteins in the feline saliva. It is widely known that some animals have toxins that disrupt hemostasis [18] and there is some experimental evidence that cat saliva composition can change with different states of sympathetic and parasympathetic stimulation [19]. Our knowledge of the differences between mammalian saliva is still limited, but recent advances have shown that there are many species-specific proteins with unclear functions [20] that, if properly characterized, can shine some light on our understanding of exogenous thrombotics.

While the association between the cat bite and the DVT might be strictly incidental, the extremely unusual association warrants documentation and further study.

## Conclusions

Although most of the cat-related injuries go unnoticed due the lack of severe complications, the clinical association of DVT and a previous history of a recent cat bite in a patient with no other known risk factors may suggest a causality between these two events.
